# Metabolic regulation of calcium pumps in pancreatic cancer: role of phosphofructokinase-fructose-bisphosphatase-3 (PFKFB3)

**DOI:** 10.1186/s40170-020-0210-2

**Published:** 2020-04-02

**Authors:** D. A. Richardson, P. Sritangos, A. D. James, A. Sultan, J. I. E. Bruce

**Affiliations:** 1 Division of Cancer Sciences, School of Medical Sciences, University Of Manchester, Michael Smith Building, Oxford Road, Manchester, M13 9PT UK; 2 Department of Biology, University of York, Heslington, York, UK

**Keywords:** Metabolism, Glycolysis, PFKFB3, Calcium pumps, PMCA, Calcium overload, Pancreatic cancer, PDAC

## Abstract

**Background:**

High glycolytic rate is a hallmark of cancer (Warburg effect). Glycolytic ATP is required for fuelling plasma membrane calcium ATPases (PMCAs), responsible for extrusion of cytosolic calcium, in pancreatic ductal adenocarcinoma (PDAC). Phosphofructokinase-fructose-bisphosphatase-3 (PFKFB3) is a glycolytic driver that activates key rate-limiting enzyme Phosphofructokinase-1; we investigated whether PFKFB3 is required for PMCA function in PDAC cells.

**Methods:**

PDAC cell-lines, MIA PaCa-2, BxPC-3, PANC1 and non-cancerous human pancreatic stellate cells (HPSCs) were used. Cell growth, death and metabolism were assessed using sulforhodamine-B/tetrazolium-based assays, poly-ADP-ribose-polymerase (PARP1) cleavage and seahorse XF analysis, respectively. ATP was measured using a luciferase-based assay, membrane proteins were isolated using a kit and intracellular calcium concentration and PMCA activity were measured using Fura-2 fluorescence imaging.

**Results:**

PFKFB3 was highly expressed in PDAC cells but not HPSCs. In MIA PaCa-2, a pool of PFKFB3 was identified at the plasma membrane. PFKFB3 inhibitor, PFK15, caused reduced cell growth and PMCA activity, leading to calcium overload and apoptosis in PDAC cells. PFK15 reduced glycolysis but had no effect on steady-state ATP concentration in MIA PaCa-2.

**Conclusions:**

PFKFB3 is important for maintaining PMCA function in PDAC, independently of cytosolic ATP levels and may be involved in providing a localised ATP supply at the plasma membrane.

## Introduction

Pancreatic ductal adenocarcinoma (PDAC) is the most common form of pancreatic cancer and with a 5-year survival rate of < 3%, it has one of the worst survival rates of all cancers [1, 2]. Currently, the only curative treatment for PDAC is surgical resection, which is only possible for a fraction of patients and is often unsuccessful [3]. PDAC is often resistant to currently available treatments and there is a clear unmet need for novel drugs to specifically target PDAC.

PDAC cells undergo a metabolic switch from predominantly mitochondrial to highly glycolytic metabolism, referred to as the Warburg effect [4]. The Warburg phenotype facilitates numerous cancer hallmarks including rapid proliferation, invasion and immune evasion [5–8]. Our recent work has identified that glycolytic ATP is required to fuel ATP-dependent plasma membrane calcium ATPases (PMCAs), responsible for maintaining low intracellular calcium ([Ca^2+^]_i_). Inhibition of glycolysis but not mitochondrial metabolism leads to attenuation of PMCA function, cytotoxic calcium overload and cell death in PDAC cells [9]. Moreover, reversal of the Warburg effect protects PMCA function in PDAC cells treated with glycolytic inhibitors [10]. Taken together, this suggests that the glycolytic dependency of PMCAs may present a novel therapeutic target in PDAC.

After identifying that a glycolytic ATP supply to PMCAs is critical for PDAC cell survival, the next logical step was to identify key glycolytic enzymes that are upregulated in PDAC and contribute to this phenotype.

Pyruvate kinase M2 (PKM2) is a splice variant of the PKM gene that is expressed in some rapidly diving cells and overexpressed in many cancers, including PDAC, and has been described as a ‘master regulator’ of the Warburg effect [11–13]. PKM2 catalyses the final step in glycolysis which produces pyruvate and generates ATP. Though cancer cells exhibit a high rate of glycolysis, PKM2 has a lower catalytic activity than PKM1; this produces a bottleneck in glycolysis which allows the build-up of glycolytic intermediates that can be utilised for anabolic processes, at the expense of ATP production [14–17].

Phosphofructokinase-1 (PFK1) is responsible for converting fructose-6-phosphate (F6P) to fructose-1,6-bisphosphate (F16BP), which is the major rate-limiting reaction in glycolysis. PFK1 is overexpressed in numerous cancers and correlates with poor prognosis in lung cancer [18–22]. However, PFK1 is inhibited by a high ATP:AMP ratio. Cancer cells have been reported to have a low intracellular ATP concentration, thus preventing inhibition of PFK1 and driving the Warburg phenotype [19]. F16BP, the product of PFK1 activity also activates pyruvate kinase M2 (PKM2), responsible for the production of pyruvate, the final step of glycolysis, thus acting to drive glycolysis further [16].

PFK1 activity can also be increased by fructose-2,6-bisphosphate (F26BP), a potent allosteric activator that drives glycolytic flux. F26BP is produced by phosphofructokinase-fructose-bisphosphatases (PFKFBs), which phosphorylate F6P to produce F26BP. PFKFBs are bifunctional enzymes that also possess phosphatase activity and can, therefore, remove phosphate from F26BP to regenerate F6P. There are four PFKFB isoforms (PFKFB1–4), of which PFKFB3 exhibits the highest kinase:phosphatase activity of all (~ 700-fold). PFKFB3 is able to produce F26BP at a high rate and thus drives the Warburg effect [23, 24]. Under hypoxic conditions, this kinase:phosphatase activity can be increased up to 3000-fold following phosphorylation at Ser460 by PKA or AMPK [25]. Hypoxia is a common feature of PDAC and hypoxia-inducible factor HIF1-α has been shown to induce expression of PFKFB3 [26].

PFKFB3 is overexpressed in numerous cancers including PDAC [27, 28]; this finding led to the development of the specific small-molecule inhibitor 3PO and then more potent 3PO derivatives: PFK15 and PFK158 [24]. PFK15 has been effective in reducing tumour cell growth in vitro and has also reduced tumour growth and metastasis in xenograft models [29–31]. Importantly, neither animal studies nor phase I trials has identified any major detrimental effects of PFK158 suggesting that there are no off-target effects and that inhibition of PFKFB3 is not ubiquitously harmful [32–34].

The present study aimed to test whether the PFKFB3, a driver of the Warburg phenotype, plays a role in providing a glycolytic ATP supply to PMCAs in PDAC.

## Materials and methods

### Bioinformatics

Oncomine software was used to generate heatmaps (Thermo Fisher Scientific, Ann Arbor, MI) and access expression data from the Badea Pancreas study [35]. Survival data was accessed and Kaplan-Meier plot generated using PROGgeneV2 (Indiana University Purdue University, Indianapolis, IN).

### Cell culture

MIA PaCa-2 and PANC1 cells were obtained from the ATCC (Manassas, Virginia, USA), whereas human pancreatic stellate cells, isolated from resected tumour tissue (HPSCs) were a kind gift from Professor David Yule (University of Rochester, New York, USA). BxPC-3 cells were a kind gift from Dr. Ayse Latif (University of Manchester, UK). All cell types were grown in 75-cm^2^ culture-treated flasks (Corning, New York, USA) in high-glucose Dulbecco’s modified Eagle’s media (DMEM) or RPMI-1640 (Sigma Aldrich, Gillingham, UK). All media was supplemented with 10% foetal bovine serum (Biowest, Nuaillé, France) and 100-units/mL penicillin and 100μg/mL streptomycin (Sigma). All flasks were kept incubated at 37 °C and in 5% CO2. MIA PaCa-2 cells were discarded after being sub-cultured 30 times whereas HPSCs were discarded after 10.

### Drug preparation

Stock solutions of specific PFKFB3 inhibitor 1-(4-Pyridinyl)-3-(2-quinolinyl)-2-propen-1-one (PFK15) were made up to 30 mM in dimethyl sulfoxide (DMSO, both Sigma). PFK15 was further diluted in DMEM for experimentation. For calcium imaging experiments, PFK15 stock solutions were diluted directly in HEPES-buffered physiological saline solution (HEPES-PSS: 138 mM NaCl, 4.7 mM KCl,10 mM HEPES, 5.5 mM glucose, 1.28 mM CaCl2, 0.56 mM MgCl2, pH 7.4). Calcium-free HEPES-PSS (0Ca-HEPES-PSS: 138 mM NaCl, 4.7 mM KCl,10 mM HEPES, 5.5 mM glucose, 1.28 mM CaCl2, 0.56 mM MgCl2, 1 mM EGTA pH 7.4) and high calcium HEPES-PSS (138 mM NaCl, 4.7 mM KCl,10 mM HEPES, 5.5 mM glucose, 20 mM CaCl2, 0.56 mM MgCl2, pH 7.4) were used for clearance assays. All drugs used in the metabolic inhibitor cocktail: iodoacetate 2 mM, (IAA), bromopyruvate 200 μM (BrPy), oligomycin 10 μM (OM) and carbonyl cyanide 3-chlorophenylhydrazone 4 μM (CCCP) were obtained from Sigma-Aldrich and stock solutions dissolved in milliQ water (IAA) or DMSO.

### Cell proliferation and viability assays

Viable cells were counted using trypan blue staining and a haemocytometer. 2500 cells (5000 BxPC-3 cells) were seeded onto clear 96-well plates (Corning). Wells containing only media and vehicle acted as blanks. Plates were then incubated for 2–96 h. At each time-point, 10 μL of CCK-8 (Dojindo, Munich, Germany) was added to each well and plates were incubated for another 60 min at 37 °C. Following incubation, the plate was read for absorbance at 450 nm using a Synergy HT plate reader, operated by Gen 5 software (both Biotek, Whiting, Vermont, USA). Absorbance from blank wells containing only DMEM and vehicle were subtracted from each well reading. Cells were then fixed using of 100 μL 10% trichloroacetic acid at 4 °C for 1 h, washed with milliQ water and dried at 50 °C. Fixed cells were stained using 100 μL 0.057% sulforhodamine-B (SRB) (Sigma) dissolved in 1% acetic acid. Unbound dye was washed off of the cells using 1% acetic acid and plates were then dried at 50 °C. Bound dye was re-suspended in 200-μL 10-mM Tris-Base solution (Sigma) and absorbance at 540 nm was read using the Synergy HT plate reader. There were 4 replicates for each condition in every experiment.

### Global ATP measurement

MIA PaCa-2 cells were seeded at 10,000 cells/well into clear-bottomed, white-walled 96-well plates (Corning) and allowed to adhere overnight. The following day, wells were treated with PFK15, inhibitor cocktail (4 μM CCCP, 10 μM oligomycin, 200 μM bromopyruvate and 2 mM iodoacetate) or vehicle (DMSO). Global ATP was quantified using a Vialight plus assay (Lonza, Slough, UK), following the manufacturer’s instructions, using a Biotek HT plate reader.

### Seahorse XFe analysis

Both extracellular acidification rate (ECAR) and oxygen consumption rate (OCR) were measured simultaneously using a Seahorse XFe96 analyser (Agilent, CA, USA). MIA PaCa-2 cells, cultured as described above, were seeded in the manufacturer’s 96-well plate at 20,000 cells/well and allowed to attach overnight. One hour before experimentation, media was changed to Seahorse XF DMEM medium pH 7.4 (Agilent), supplemented to contain 25 mM glucose, 4 mM L-glutamine and 2 mM pyruvate. The plate was allowed to equilibrate for 1 h in a CO_2_-free incubator at 37 °C before loading into the Seahorse analyser. Seahorse XF96 cartridges were hydrated as per the manufacturer’s instructions. Cells were pre-treated with either PFK15 or vehicle 20 min before the plate was loaded onto the analyser.

### Immunoblotting

Cells were seeded onto 6-well plates (Corning) and allowed to adhere overnight. After treatment, cells were lysed with ice-cold lysis buffer (50 mM Tris-base, 40 mM sodium pyrophosphate,100 μM sodium fluoride, 150 mM sodium chloride, 1% Triton X-100, 10 mM EGTA and EDTA sodium salt, 1x complete EDTA-free protease inhibitor, 1× phostop (Roche, Hertfordshire, UK). Lysates were subjected to sonication at an amplitude of 15 μm using a soniprep-150 sonicator (Sanyo, Watford, UK). Protein was quantified using a BCA assay kit and Nanodrop 2000 (both Thermo Fisher, Waltham, Massachusetts, USA).

Anti-PFKFB3, GAPDH and anti-PARP monoclonal rabbit antibodies were used at a concentration of 1:1000 for probing (#13123, #5174 and #9542S respectively Cell Signaling Technology). Mouse anti-PMCA4 (JA9, # MA1–914, Thermofisher) was also used 1:1000. HRP conjugated goat anti-rabbit antibody (#7074 Cell Signalling Technology) or HRP conjugated horse anti-mouse (#7076 Cell Signalling Technology) were used as a secondary antibody. Clarity ECL, a Chemidock and ImageLab acquisition software were used to produce digital images (BioRad). ImageJ software was used to quantify an PARP:cleaved-PARP band volumes in MIA PaCa-2, BxPC-3 and HPSCs after 6 h of treatment with 10 μM PFK15.

### Membrane protein isolation

Membrane and cytosolic proteins were separated using a plasma membrane protein extraction kit (Abcam, #65400). Approximately 2 × 10^8^ cells were grown in 160-mm plates and were treated as per the manufacturer’s instructions. Both membrane and cytosolic fractions were then assessed by Immunoblot.

### Calcium imaging

MIA PaCa-2 cells and HPSCs were seeded onto 16-mm or 25-mm glass coverslips in non-treated 6-well plates (Corning) containing 25 mM glucose DMEM. These cells were then incubated for 24 h to allow adherence to the coverslip. Cells were then loaded with 5 μM Fura-2-AM (Teflabs, Austin, Texas, USA) as previously described [9]. Two microscopes were used for imaging, a Nikon Diaphot and a Nikon TE2000S and images were obtained using an Orca CCD (Hamamatsu, Hamamatsu, Shizuoka, Japan) CoolSNAP HQ progressive-scan CCD camera (Roper Scientific, Sarasota, Florida, USA) respectively. Excitation was performed using a monochromator illumination system (Cairn Research, Faversham, UK). All image acquisition was controlled by Metafluor software (Molecular Devices, San Jose, California, USA), including both excitation and data recording. Cells were perfused with either standard HEPES-PSS (138 mM NaCl, 4.7 mM KCl, 1.28 mM CaCl2, 0.56 mM MgCl2, 5.5 mM glucose, 10 mM HEPES, pH 7.4), or HEPES-PSS containing a drug, using a gravity-fed perfusion apparatus (Harvard Apparatus, Holliston, Massachusetts). Calcium overload and clearance experiments were conducted as previously described as was calcium calibration [9, 10, 36].

### Data analysis

All data analysis was conducted using Prism 7 software (GraphPad) and Microsoft Excel (Microsoft). Data was tested for normal distribution using a Kolmogorov-Smirnoff test and the outcome determined whether a parametric or non-parametric statistical test was used and a *p* value below 0.05 was considered significant.

## Results

### PFKFB3 is overexpressed in PDAC and predicts poor prognosis

**Fig. 1:**
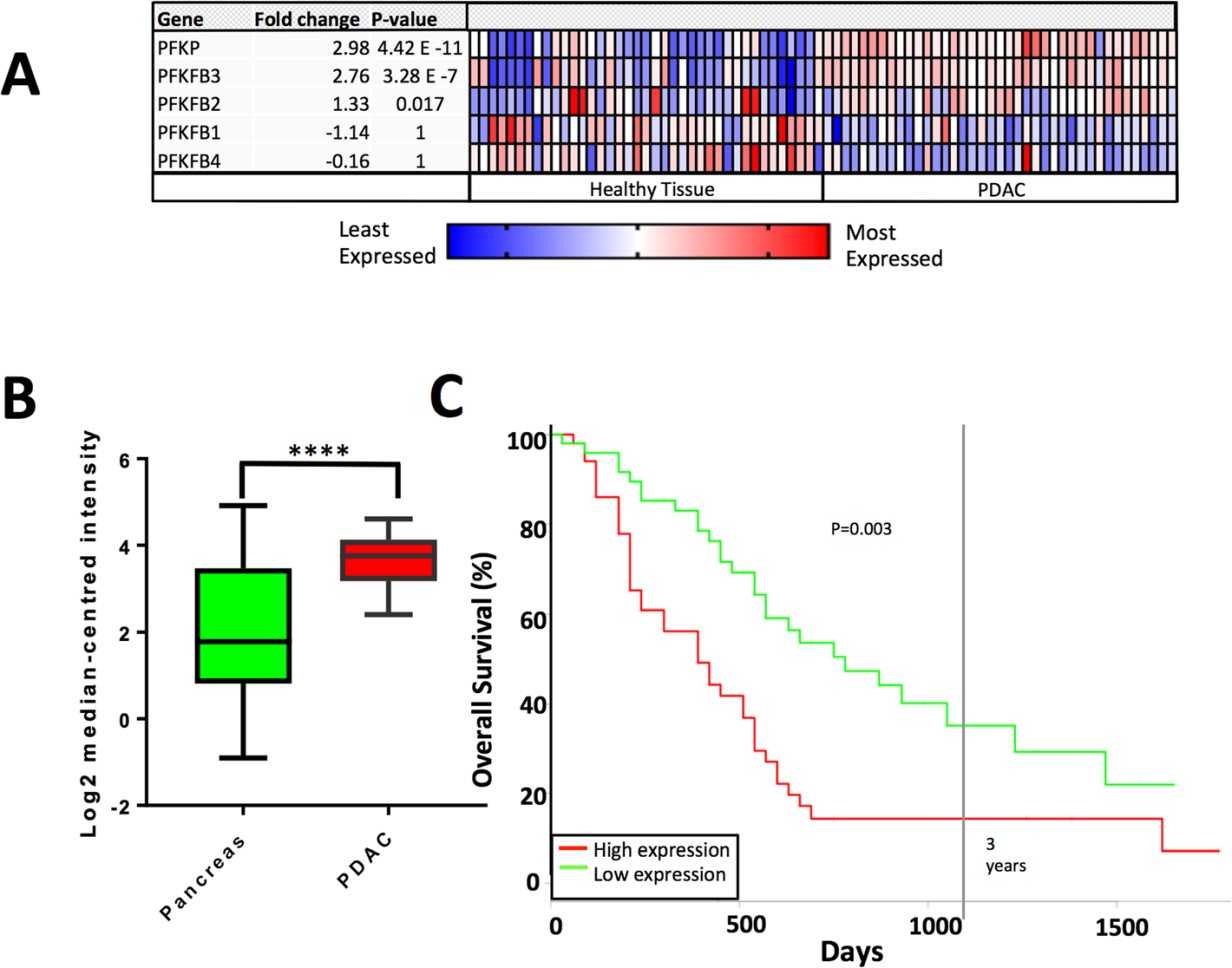
Expression of PFKFB3 is upregulated and correlates with poor prognosis in PDAC: **a** Heat map analysis of overexpression of PFKFB isoforms 1–4 and PFKP in tumour tissue versus healthy pancreatic tissue in the pancreas resected from PDAC patients (datamined from Badea Pancreas dataset using Oncomine [35]) **b** Box and whisker plot displaying PFKFB3 expression in tumour tissue versus healthy tissue from resected tissue (unpaired *t* test; *n* = 39). **c** Survival rates of PDAC patients with high or low expression of PFKFB3 estimated using the Kaplan–Meier method and proportional hazards analysis; *n* = 51, *p* = 0.003 (figure generated using PROGgeneV2, ****p < 0.0005)

Expression of PFKFB1–4 and PFKP in tumour tissue versus healthy pancreatic epithelia was assessed by interrogating the Oncomine database (www.oncomine.com, Thermo Fisher Scientific, Ann Arbor, MI) and array dataset: Badea Pancreas [35]. This demonstrated that PFKFB3 and PFKP were overexpressed in tumour tissue, whilst there was modest upregulation of PFKFB2 and expression of PFKFB1 and PFKFB4 was downregulated, compared with healthy tissue from the same pancreatic resection (*n* = 39, *p* < 0.0001, Fig. 1a/b). Next, the relationship between PFKFB3 expression and overall survival was assessed using expression data from 51 PDAC patients using the PROGgeneV2 software [37, 38], Indiana University Purdue University, Indianapolis, IN) and survival data from PFKFB3 expression was bifurcated at the median and survival was assessed using the Kaplan-Meier method and PROGgeneV2’s in-built proportional hazards analysis. Patients with low PFKFB3 expression lived significantly longer than patients with high PFKFB3 expression (hazard ratio = 1.64, *p* = 0.0033; Fig. 1c).

### PFK15 reduces cell proliferation and induces cell death in PDAC MIA PaCa-2 cells

**Fig. 2:**
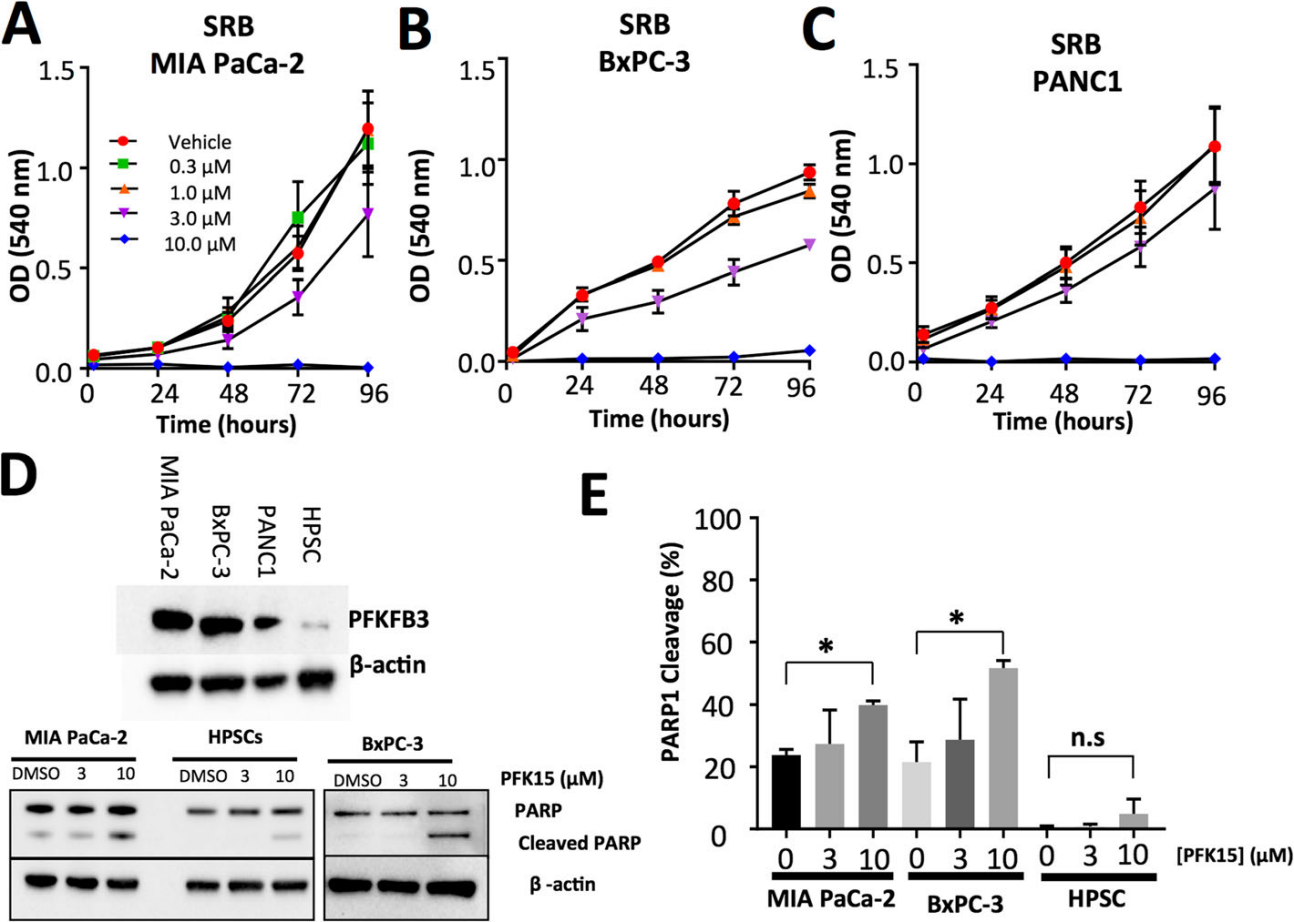
PFK15 reduces proliferation and induces death in MIA PaCa-2 but not human pancreatic stellate cells: PDAC cell lines:MIA PaCa-2, BxPC-3 and PANC1 were treated with 0.3–10 μM PFK15 for up to 96 h; cell proliferation was measured using a sulforhodamine-B assay (**a-c**). Western blot for PFKFB3 in MIA PaCa-2, BxPC-3 and human pancreatic stellate cells (HPSCs) (**d**i). Western blot for PARP cleavage (**d**ii)**,** cells were treated with PFK15 (3–10 μM) or vehicle (DMSO) for 6 h and bands were quantified; the intensity of cleaved PARP is expressed as a percentage of total PARP (**e**). Points and bars represent the mean ± SEM of 5 separate experiments. Kruskall-Wallis test; *p* < 0.05*

Glycolytic ATP is required for PMCA function; inhibition of glycolysis using iodoacetate (IAA) and bromopyruvate (BrPy) causes cytotoxic calcium overload and cell death in PDAC cells [9, 10]. To assess whether PFKFB3, a known driver of the Warburg phenotype, is required for this phenomenon, the specific PFKFB3 inhibitor PFK15 was used. The effects of PFK15 on cell proliferation and cell viability were assessed in PDAC cell lines MIA PaCa-2, BxPC3 and PANC1 using a sulforhodamine-B (SRB) assay (Fig. 2a-c) and tetrazolium-based cell counting kit (CCK8, Additional file 1: Figure S1A-C). In SRB assays, 10 μM PFK15 produced a significant reduction in MIA PaCa-2 cell proliferation after 72 h (Fig. 2a). However, the SRB assay is limited by numerous wash steps that can lead to loss of poorly adhered cells, and thus underestimation of cell number. Therefore, any drug that affected cell adhesion, independent of proliferation, would compromise the validity of conclusions drawn. To address this, we used an accompanying CCK8 assay, which measures the redox potential of live cells, independent of cell adherence. Results from CCK8 assays closely mirrored SRB results indicating that changes observed were real (Fig. 2b).

In both of these assays, it is difficult to distinguish between reduced proliferation and cell death and, based on the steep concentration-effect relationship observed, it is possible that 10 μM PFK15 induces cell death. Therefore, apoptosis was assessed by immunoblot for poly (ADP-ribose) polymerase (PARP), a cleavage target of caspase 3 during apoptosis [39, 40]. PARP cleavage is a marker of apoptosis and thus the ratio of cleaved: whole PARP was assessed in MIA PaCa-2, BxPC-3 and human pancreatic stellate cells (HPSCs), as a non-cancer control with much lower PFKFB3 expression (Fig. 2di). Treatment with 10 μM PFK15 for 24 h caused a significant increase in PARP cleavage in MIA PaCa-2 and BxPC-3 when compared with vehicle-treated cells (Kruskall-Wallis test: MIA PaCa-2 vehicle: 25.07 ± 1.91, 10 μM PFK15: 40.46 ± 1.19 *p* = 0.032, *n* = 5, BxPC3 vehicle: 21.51 ± 6.57, 10 μM PFK15: 51.80 ± 2.44, *n* = 3 *p* = 0.016 Fig. 2dii/e). MIA PaCa-2 cells were selected going forward, these cells have high PFKFB3 expression, high growth rate and have been identified as highly glycolytic in comparison to other PDAC cell lines; they also express mutant p53 and mutant K-RAS, the most common mutations in PDAC [9, 10, 41–43].

### PFK15 produces calcium overload in MIA PaCa-2 but not human pancreatic stellate cells

**Fig. 3:**
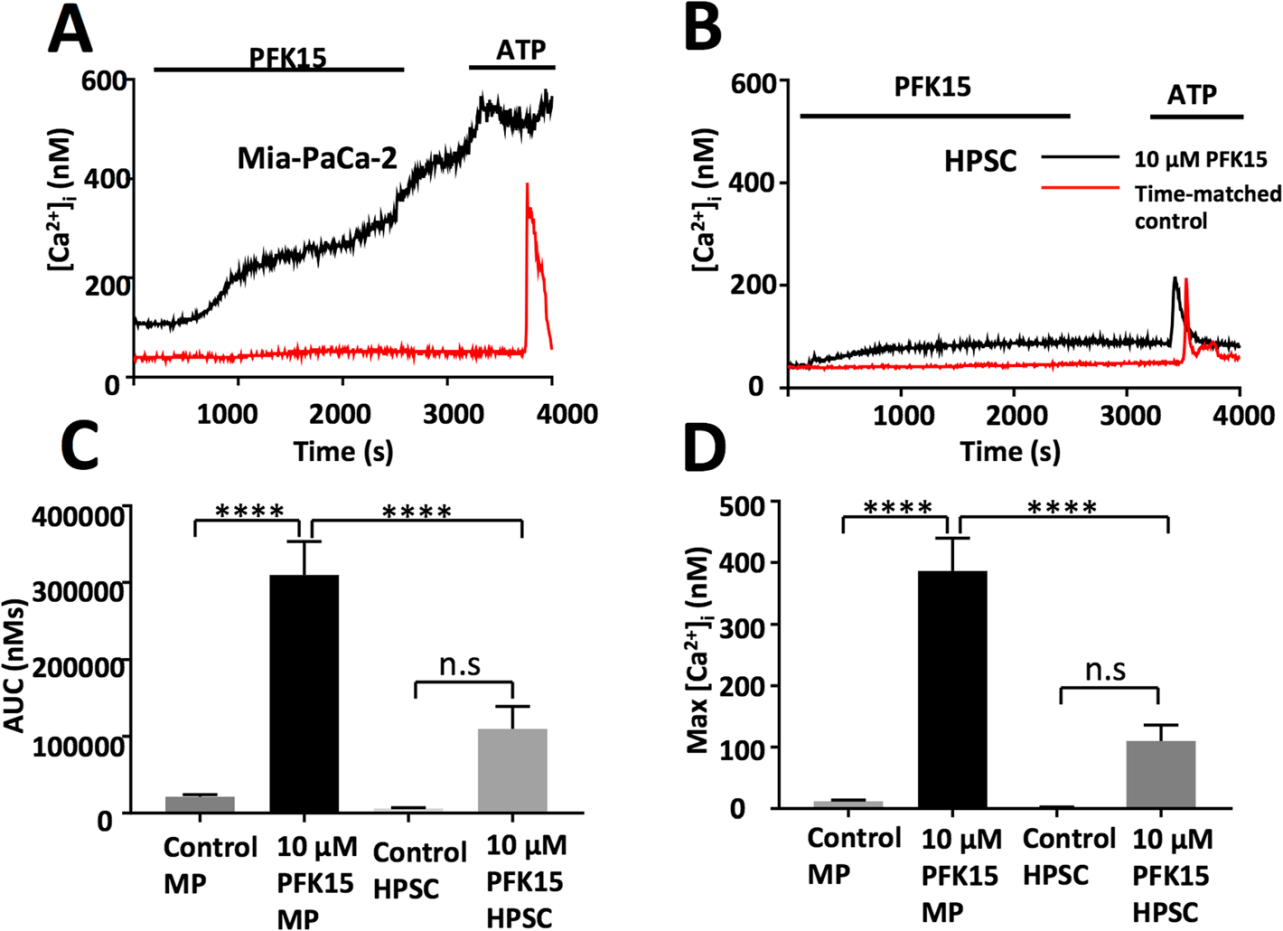
PFK15 causes calcium overload in MIA PaCa-2 but not HPSC: Fura-2 fluorescence microscopy was used to assess the effects of PFK15 on intracellular calcium ([Ca^2+^]_i_) in MIA PaCa-2 cells (**a**) and human pancreatic stellate cells (HPSCs) (**b**). Cells were loaded with fura-2 dye and perfused with HEPES-PSS or HEPES-PSS containing 10 μM PFK15 for 40 min then HEPES-PSS containing 100 μM ATP. Traces were assessed for area under the curve (**c**) and maximum calcium increase (**d**). **a** and **b** are representative traces for MIA PaCa-2 and HPSCs respectively, **c** and **d** represent the mean ± SEM, *n* = 3–12 separate experiments, one-way ANOVA, ****p < 0.0005

Previously, glycolytic inhibitors have been shown to produce cytotoxic calcium overload in PDAC cell lines [9]. We sought to assess whether this was the case for specific PFKFB3 inhibitor PFK15. Fura-2 loaded MIA PaCa-2 cells were perfused with HEPES-buffered physiological saline solution (HEPES-PSS) with or without 10 μM PFK15 for 40 min, during which [Ca^2+^]_i_ was measured using fura-2 imaging. As expected, untreated MIAPaCa-2 cells were able to maintain [Ca^2+^]_i_ at a relatively constant resting level for the duration of the experiment, however [Ca^2+^]_i_, increased significantly after PFK15 treatment (*p* < 0.0001; Fig. 3). To determine whether this effect was due to PFKFB3 inhibition, HPSCs, which have very low PFKFB3 expression, were assessed in the same way and no significant calcium overload was observed (Fig. 3).

### PFK15 inhibits PMCA activity in MIA PaCa-2 but not human pancreatic stellate cells

**Fig. 4:**
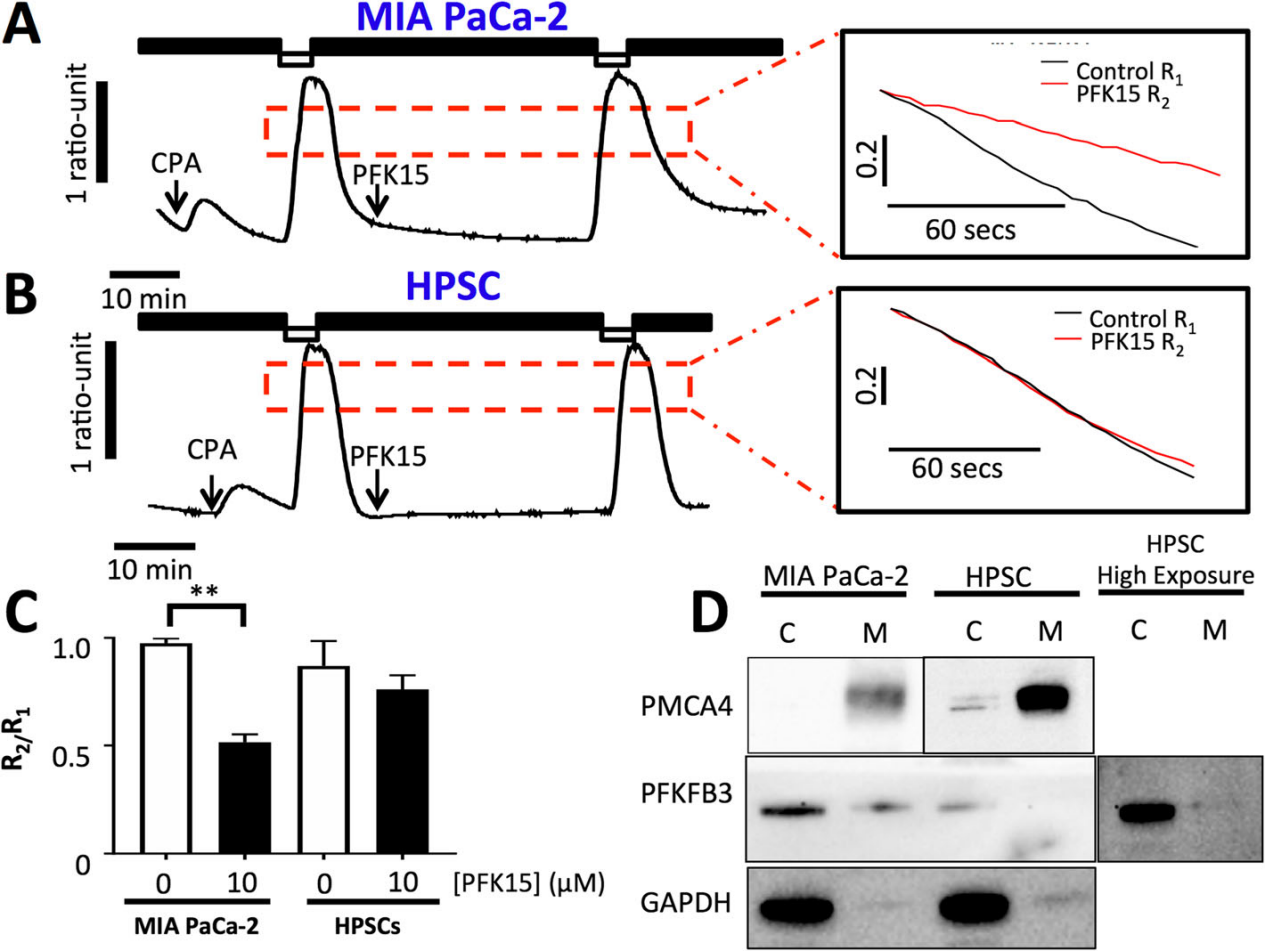
PFK15 causes PMCA inhibition in MIA PaCa-2 but not human pancreatic stellate cells: PMCA activity was measured in MIA PaCa-2 cells and HPSCs perfused under constant SERCA blockade (cyclopiazonic acid, CPA, 30 μM) and calcium-free conditions and 1 mM EGTA (black bar). Subsequent ER Ca^2+^ depletion caused store-operated calcium entry when 20 mM Ca^2+^ was introduced (white bar) calcium efflux was used as a measure of PMCA activity before and after 30 min incubation with or without 10 μM PFK15. Representative traces showing clearance experiments with PFK15 in MIA PaCa-2 (**a**) and HPSCs (**b**). The rate of clearance was calculated for the fastest 2 min of the first clearance phase (*R*_1_), the clearance rate from the same starting ratio was then calculated in the second clearance phase (*R*_2_). *R*_1_/*R*_2_ was calculated for both MIA PaCa-2 and HPSC with and without PFK15 treatment (**c**). Membrane and cytosolic protein samples from MIA PaCa-2 cells were produced using a membrane protein extraction kit and PFKFB3 levels assessed by immunoblot. PMCA4 was used as a positive control for membrane samples and GAPDH was used as a positive control for cytoplasmic samples, a representative image is shown (**d**). Bars represent the mean ± SEM of 3–5 experiments. Kruskall-Wallis test; **p < 0.005

Previously, the effect of glycolytic inhibition on PMCA function has been assessed following functional and pharmacological isolation of PMCA activity [9, 10]. Cells were subjected to ER calcium depletion using SERCA inhibitor cyclopiazonic acid (CPA, 30 μM) throughout the experiment, in calcium-free HEPES-PSS containing 1 mM EGTA (0Ca-HEPES-PSS). Store-operated calcium entry occurs when perfusion is switched to 20 mM calcium HEPES-PSS; when calcium is removed and the clearance is mediated by PMCAs [9, 10, 44, 45]. This is repeated twice per experiment and the clearance rate of the fastest 2-min portion of the first clearance phase (R_1_) is compared with the second clearance rate from the same fura-2 ratio (R_2_), clearance is assumed to be linear during this phase; however, any trace that produced an R-squared value below 0.9 was excluded. This method was employed to test the effects of PFK15 and distinguish whether PMCA inhibition was responsible for PFK15-induced calcium overload. Both MIA PaCa-2 and HPSCs were able to conserve PMCA-mediated clearance with 30 min of 0Ca-HEPES-PSS between clearance phases, in time-matched controls; producing mean R_2_/R_1_ values of 0.98 ± 0.02 (*n* = 5) and 0.87 ± 0.11 (*n* = 3) respectively. When 10 μM PFK15 was added for 30 min between clearance phases, a significant reduction of clearance rate was observed in MIA PaCa-2 but not HPSCs when compared with time-matched controls (R_2_/R_1_ = 0.52 ± 0.04 (*n* = 5) and 0.76 ± 0.06 (*n* = 4) respectively, Fig. 4a-c).

### PFKFB3 is localised to the plasma membrane in PDAC

In order to determine whether a pool of PFKFB3 is localised to the plasma membrane, in close proximity to PMCAs, a plasma membrane protein isolation kit was employed and samples were assessed by immunoblot. This kit is designed to specifically isolate plasma membrane proteins using a series of spin steps and produces lysates with over 90% purity. Membrane samples from MIA PaCA-2 cells and HPSCs were verified using PMCA4 as a positive control whilst GAPDH was used as a cytosolic control (Fig. 4d). As expected, PFKFB3 was again shown to be much lower in HPSCs compared with MIA PaCa-2 cells. PFKFB3 was identified in the plasma membrane fraction of MIA PaCa-2 but not HPSCs. Even when the membrane was overexposed, PFKFB3 was not detected in the membrane of HPSCs (Fig. 4d).

### PFK15 reduces glycolysis and mitochondrial respiration but does not affect global ATP levels in PDAC

**Fig. 5:**
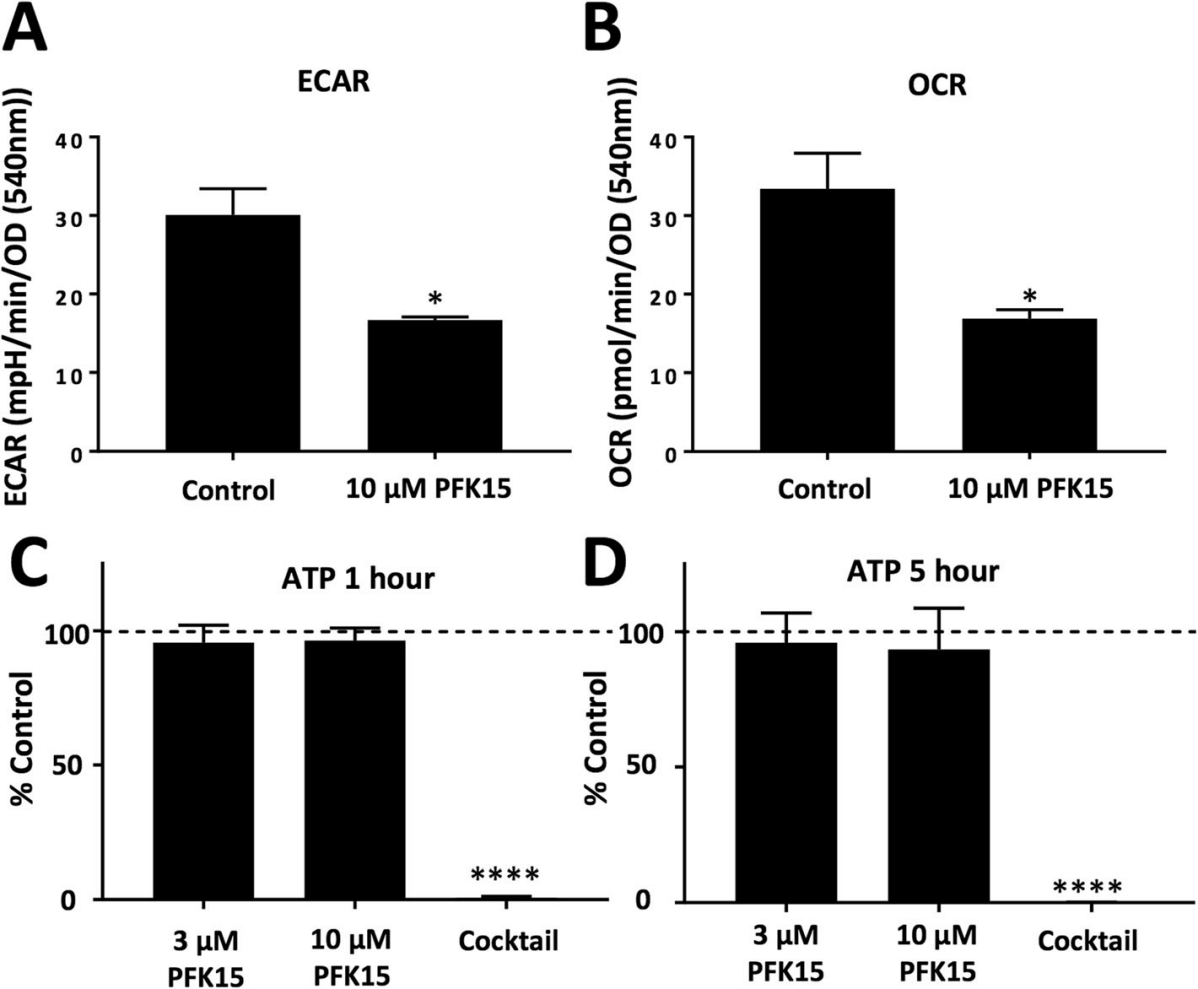
PFK15 reduces both ECAR and OCR but does not cause ATP depletion in MIA PaCa-2 cells: **a**, **b**: MIA PaCa-2 cells were treated with 10 μM PFK15 or vehicle (DMSO) for 20 min before loading onto a seahorse XFe analyser. Extracellular acidification rate (ECAR, **a**) and oxygen consumption rate (OCR, **b**) were recorded after 5 min. MIA PaCa-2 cells were treated for 1 (**c**) or 5 (**d**) h with PFKFB3 inhibitor PFK15 (3 or 10 μM), or a cocktail containing 4 μM CCCP, 10 μM oligomycin, 200 μM bromopyruvate and 2 mM iodoacetate as a positive control. ATP was measured using a luciferase-based assay kit and replicates were averaged and normalised to vehicle-treated, time-matched control. Data presented are the mean ± SEM, *n* = 4 separate experiments, each containing 4–8 replicates. Unpaired *t* test/ Kruskall-Wallis test; *p < 0.05

In order to assess whether the effects of PFK15 on cell proliferation and [Ca^2+^]_i_ in MIA PaCa-2 cells were due to metabolic inhibition, the effects on glycolysis (extracellular acidification, ECAR) and mitochondrial metabolism (oxygen consumption, OCR) were assessed using a Seahorse XFe analyser. Due to solubility issues with injection, MIA PaCa-2 cells were pre-treated with 10 μM PFK15 for 20 min and then ECAR and OCR were measured, simultaneously. Cells treated with PFK15 showed significantly lower steady-state ECAR and OCAR at all time points compared to control (unpaired *t* test *p* < 0.05, *n* = 3). Both ECAR and OCR remained stable in control cells for the duration of the experiment (Fig. 5a/b).

Next, we wanted to test whether this change in ECAR/OCR translated to ATP depletion. This was assessed using a luciferase-based assay kit. MIA PaCa-2 cells were treated with PFK15 (3 μM, 10 μM), ATP depletion cocktail (4 μM CCCP, 10 μM oligomycin, 200 μM bromopyruvate and 2 mM iodoacetate) or vehicle (DMSO) for 1–6 h. Based on the results of calcium overload experiments, it was hypothesised that 1 h would allow enough time for the drug to exert any effects on ATP that subsequently cause a cytotoxic calcium overload. Surprisingly, PFK15 caused no significant global ATP depletion even after 1 or 5 h of PFK15 treatment, whereas the ATP depletion cocktail caused close to 100% depletion in all experiments (Fig. 5, *n* = 4).

## Discussion

The present study is the first to demonstrate a link between PFKFB3 and calcium homeostasis in PDAC and supports the hypothesis that targeting glycolysis is a viable treatment in PDAC. Glycolytic driver PFKFB3, which has the highest kinase activity of the PFKFB family, is overexpressed in PDAC, whereas PFKFB isoforms which oppose aerobic glycolysis are downregulated (Fig. 1). We have identified that the PFKFB3 inhibitor, PFK15, causes cytotoxic calcium overload via inhibition of PMCA function, ultimately leading to cell death. This effect was not observed in non-cancerous HPSCs. This is of therapeutic importance as any new therapeutic strategy for PDAC must be specific. PFK15 has previously been effective in reducing tumour growth when administered to athymic mice with PDAC (BxPC-3), glioblastoma (U-87 MG) and colon adenocarcinoma (CT26) xenografted tumours. Importantly, PFK15 did not have severe side effects, despite PFKFB3 expression in the muscle, adipose tissue and other tissues [24, 46]. The present study provides further evidence to support the hypothesis that PFK15 has the potential to be used as an anti-cancer drug in PDAC.

Previous work leading to this study demonstrated that glycolytic inhibitors, but not mitochondrial inhibitors cause significant global ATP depletion and the consequent inhibition of PMCA activity and calcium overload [9, 10]. In the present study, PFK15 induced inhibition of the PMCA, calcium overload and cell death in PDAC cells, without any effect on steady-state global ATP. Moreover, in Seahorse XF experiments PFK15 reduced both ECAR, which is expected for a bona fide glycolytic enzyme inhibitor, but also reduced OCR, which was more unexpected as one would expect that a glycolytic inhibitor would cause a compensatory increase in OCR in an attempt to preserve cellular ATP. Although we cannot rule out that PFK15 may have previously unreported non-specific effects on mitochondrial function, it is important to emphasise that our previous studies have shown that mitochondrial inhibitors, which cause profound inhibition of OCR, do not affect cell survival/growth, reduce ATP or inhibit PMCA activity [9, 10]. This, therefore, suggests that the effects of PFK15 on PMCA activity are due to inhibition of glycolysis, rather than mitochondrial function. Nevertheless, the effect of PFK15 on ECAR and OCR without affecting steady-state ATP presents a functional paradox which is difficult to reconcile. However, a possible explanation is that the steady-state ATP concentration within the cell is dependent not only on the ATP production rate but also on the ATP consumption rate. Given that PFK15 inhibits cell proliferation and is reported to arrest the cell cycle [26, 30]; which are major ATP consuming processes, it is possible that reduced ATP production, due to inhibition of glycolysis, coincides with a corresponding reduced ATP consumption; such that steady-state ATP is maintained. Furthermore, in the context of inhibition of the PMCA, it is also possible that PFK15 inhibits a significant pool of PFKFB that resides in close proximity to the PMCA at the membrane, where it provides a privileged ATP supply to the PMCA without necessarily affecting global steady-state ATP.

Indeed, in the present study, PFKFB3 was identified in the plasma membrane fraction, indicating localisation of a significant pool of PFKFB3 at the plasma membrane. This further supports the above hypothesis that PFK15 inhibits this pool of PFKFB3, thereby reducing glycolytic flux and thus localised supply of ATP to the PMCA. ATP is lower in cancer cells than in healthy tissues due to the Warburg effect and high expression of PKM2. PKM2 is overexpressed in PDAC but has low catalytic activity. This causes a bottleneck in glycolysis that allows the accumulation of glycolytic intermediates, which are utilised for anabolic processes, at the expense of ATP production [12, 14–17]. It, therefore, makes teleological sense that glycolytic enzymes, as the major source of ATP in cancer cells, are localised to where ATP is required; in close proximity to ion pumps at the plasma membrane. Ion pumps such as PMCAs and the Na^+^/K^+^-ATPase are major ATP consumers in all cells, including cancer cells. This localisation would also facilitate glycolytic flux as ATP would be consumed by ion pumps soon after production. This would keep ATP concentration below the inhibitory threshold of PFK1, thereby maintaining glycolytic flux. Such localisation has been previously described in erythrocytes, which have no mitochondria and therefore exclusively require glycolytic ATP to fuel PMCAs [47]. Experiments using plasma membrane vesicles from pig smooth muscle have found that PMCA function is maintained when glycolytic substrates are provided, indicating that glycolytic enzymes can associate with the membrane in mammalian cells [48]. Moreover, the platelet isoform of PFK1 (PFKP) is recruited to the membrane by activated EGFRs and is overexpressed in PDAC (Fig. 1a) [22], whilst the liver isoform of PFK1 (PFKL) has been found to oligomerize to form filaments which were enriched at the plasma membrane [49].

Caveolae are caveolin-1 rich invaginations in the plasma membrane, in which proteins are held in close proximity allowing close-communication between signalling complexes and can also function to assist endocytosis and exocytosis [50, 51]. PMCAs are found in caveolae, which allows regulation of PMCA activity and may allow association of PMCAs with glycolytic enzymes [52–56]. Enolase, a glycolytic enzyme has been reported to localise to the plasma membrane and in particular, caveolae [57]. Therefore, caveolae may facilitate the assembly of a glycolytic ‘metabolon’ (complex of enzymes) at the membrane and in close proximity to PMCAs. Membrane association of glycolytic enzymes could explain why glycolytic but not mitochondrial inhibitors affect PMCA function. The ATP demands of membrane-bound pumps such as PMCAs and Na^+^/K^+^ ATPases are so high that the source of ATP must be in close proximity in order to maintain function. Increasing the ATP demand of Na^+^/K^+^ ATPases causes an increase in glycolysis but not OXPHOS, whilst inhibition of Na^+^/K^+^ ATPases has the opposite effect on glycolysis [58]. Castro et al. have also demonstrated that membrane pumps have a local ATP supply in HeLa cells and that, when ATP is low, it is preferentially used by Na+/K+ ATPase pumps, leading to impaired PMCA activity. Inhibition of Na+/K+ ATPase pumps using ouabain rescues this effect. The authors described this as ‘ATP steal’. In relation to the present study, this shows that PMCAs can be inhibited by sub-maximal ATP depletion and may be highly sensitive to localised ATP depletion [59]. PMCAs also have a low-affinity ATP binding site, where ATP binding increases PMCA activity. In cell-free assays, the reported K_m_ = 330 μM [60, 61]. This value is likely to be higher in vitro*.* Taken together, this suggests that cancer cells may be much more vulnerable to PMCA inhibition than healthy cells.

Glycolytic ATP fuelling of PMCAs presents a novel therapeutic target for the treatment of cancer. As PMCAs are ubiquitously expressed, they cannot be targeted directly as this would have adverse effects on healthy tissues. However, glycolysis provides a ‘preferential’ ATP supply to PMCAs in PDAC. This may be due to association or co-localisation of glycolytic enzymes with PMCAs and/or the plasma membrane, providing a privileged ATP supply to fuel PMCAs, maintaining PMCA activity which is critical for cell survival.

In summary, PFKFB3 is overexpressed in PDAC where a pool is located at the plasma membrane. Inhibition of PFKFB3 in the PDAC cell line MIA PaCa-2 causes inhibition of PMCA function, which leads to cytotoxic calcium overload and cell death. These effects are independent of global ATP. Importantly, these effects are not observed in non-cancerous HPSCs suggesting that this phenomenon is cancer-specific. Taken together, this study describes a phenomenon that could represent a future therapeutic target for PDAC and other highly glycolytic cancers and also provides further evidence for PFK15 as an anti-cancer drug. Further understanding of this phenomenon may bring to light more novel therapeutic targets for the treatment of PDAC and other cancers.

## Conclusions

PFKFB3 is overexpressed in PDAC where a pool is located at the plasma membrane. Inhibition of PFKFB3 in the PDAC cell line MIA PaCa-2 causes inhibition of PMCA function, which leads to cytotoxic calcium overload and cell death. These effects are independent of global ATP. Importantly, these effects are not observed in non-cancerous HPSCs suggesting that this phenomenon is cancer-specific. Taken together, this study describes a phenomenon that could represent a future therapeutic target for PDAC and other highly glycolytic cancers and also provides further evidence for PFK15 as an anti-cancer drug. Further understanding of this phenomenon may bring to light more novel therapeutic targets for the treatment of PDAC and other cancers.

## Supplementary information

**Additional file 1:** **Figure S1.** PFK15 reduces cell proliferation and induces cell death in PDAC cells but not human pancreatic stellate cells.

## Data Availability

All analysed and derivative raw data available on request.

## Abbreviations

[Ca^2+^]_i_: Intracellular calcium concentration
ATP: Adenosine triphosphate
BrPy: Bromopyruvate
CaM: Calmodulin
CCK-8: Cell counting kit 8 (dojindo)
CPA: Cyclopiazonic acid
F16BP: Fructose-1,6-bisphosphate
F26BP: Fructose-2,6-bisphosphate
F6P: Fructose-6-phosphate
GLUT1: Glucose transporter 1
HPSC: Human pancreatic stellate cell (cell line)
IAA: Iodoacetate
OM: Oligomycin
OXPHOS: Oxidative phosphorylation
PARP: Poly-ADP-ribose polymerase
PDAC: Pancreatic ductal adenocarcinoma
PFK: Phosphofructokinase
PFKFB: Phosphofructokinase-fructose-bisphosphatase
PK: Pyruvate kinase
PMCA: Plasma membrane calcium ATPase
PSS: Physiological saline solution
SRB: Sulforhodamine-B
