# Supplementary material for: Metabolic regulation of calcium pumps in pancreatic cancer: role of phosphofructokinase-fructose-bisphosphatase-3 (PFKFB3)
Source: Cancer Metab. 2020 Apr 2;8:2. doi: 10.1186/s40170-020-0210-2 (PMC7114799; doi:10.1186/s40170-020-0210-2)
Supplement: Supplementary file 1 — Additional file 1: Figure S1. PFK15 reduces cell proliferation and induces cell death in PDAC cells but not human pancreatic stellate cells. [file 40170_2020_210_MOESM1_ESM.docx]

**Metabolic regulation of calcium pumps in pancreatic cancer: role of phosphofructokinase-fructose-bisphosphatase-3 (PFKFB3)**

**Richardson DA, Sritangos P, James AD, Sultan A, Bruce JIE*.**

**Daniel Richardson, Jason I.E Bruce, Ahlam Sultan, Pishyaporn Sritangos** – Division of Cancer Sciences, School of Medical Sciences, University Of Manchester, Michael Smith Building, Manchester, M13 9PT

**Andrew D James –** Department of Biology, University of York, Heslington, York

* Address for correspondence: Division of Cancer Sciences, School of Medical Sciences, University Of Manchester, Michael Smith Building, Oxford Road, Manchester, M13 9PT. Email: [jason.bruce@manchester.ac.uk](mailto:jason.bruce@manchester.ac.uk)


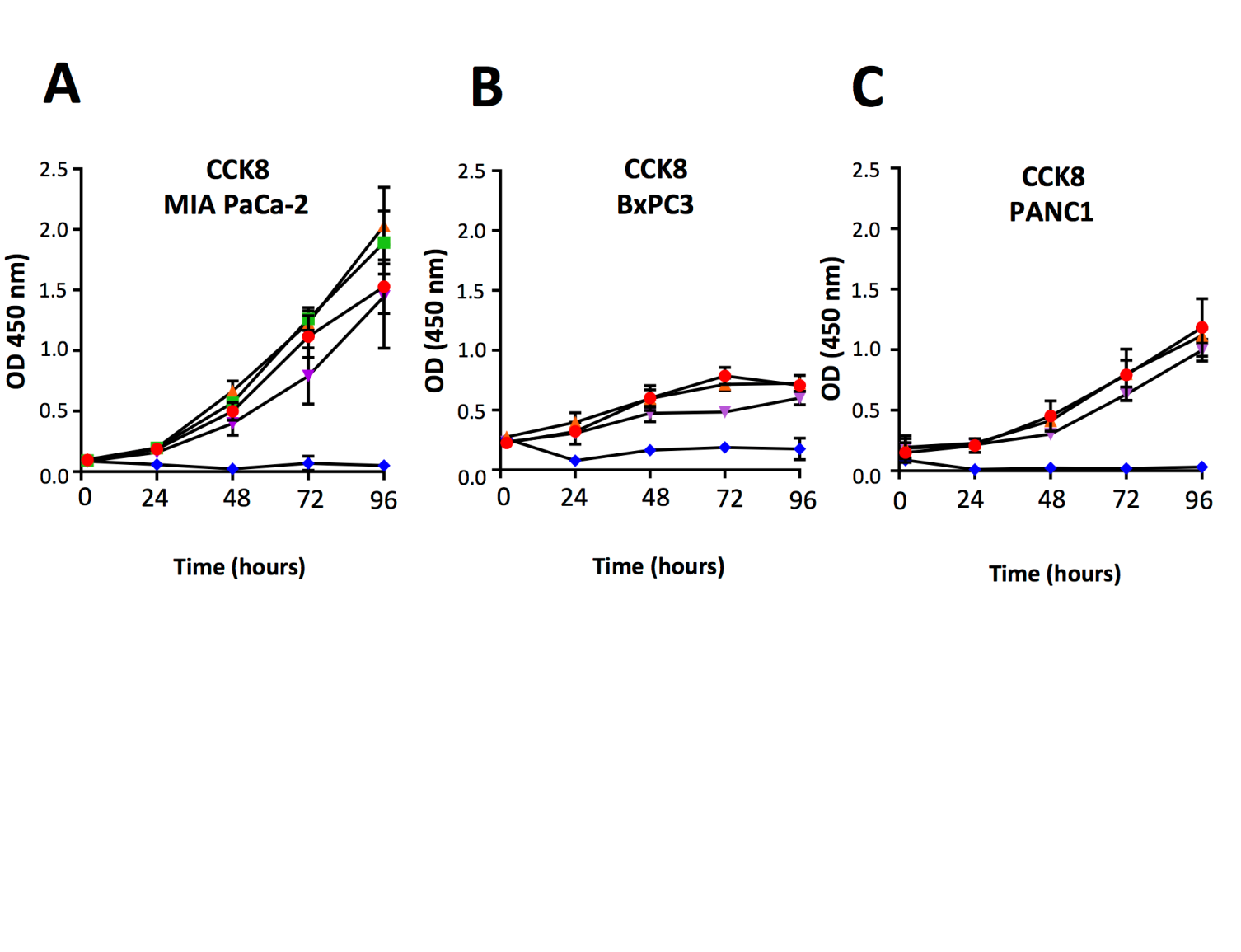


**Figure S1: PFK15 reduces cell proliferation and induces cell death in PDAC cells but not human pancreatic stellate cells:** MIA PaCa-2, BxPC-3 and PANC1 cells were treated with 0.3-10 µM PFK15 for up to 96 hours; cell proliferation was measured using a CCK8 assay (A-C). Points represent the mean ± SEM of 5 separate experiments. Kruskall-Wallis test; P<0.05 *.
